# Unveiling the Future of Oncology and Precision Medicine through Data Science

**DOI:** 10.3390/ijms25115797

**Published:** 2024-05-26

**Authors:** Apostolos Zaravinos

**Affiliations:** 1 Department of Life Sciences, School of Sciences, European University Cyprus, 2404 Nicosia, Cyprus; a.zaravinos@euc.ac.cy; 2 Cancer Genetics, Genomics and Systems Biology Laboratory, Basic and Translational Cancer Research Center (BTCRC), 1516 Nicosia, Cyprus

Information generated via next-generation sequencing (NGS) technologies is often termed multi-omics data. This includes information on DNA, RNA, protein, and epigenetic modifications, among others. Using data science in cancer genomics provides a better understanding of the molecular basis of different cancers, enabling the exploitation of this information to match each patient with the most appropriate molecularly targeted therapy, widely known as “precision medicine” [1,2]. While traditional chemotherapy and radiation treatments target cellular processes common to both healthy and cancerous cells, precision medicine directs newly developed treatments, specifically to cancer cells, based on their underlying molecular profile.

The field of oncology and precision medicine is rapidly evolving, fueled by the development of new laboratory and computational technologies for interrogating the molecular and cellular traits of cancer. In this setting, the integration of data science has become a cornerstone for advancing our understanding and treatment of cancer [3]. This Special Issue, entitled “Data Science in Cancer Genomics and Precision Medicine”, presents a collection of seven cutting-edge studies that characterize the synergy between data science and oncological research. These contributions not only showcase new findings, but also indicate the path toward personalized cancer care, underscoring the essential role of big data, genomic analysis, and computational biology in transforming patient outcomes.

The first publication of this Issue, submitted by Kraus et al., presents an unprecedented examination of how precision medicine can be used to more effectively combat HPV-related gynecological malignancies. In cervical cancer treatments, several targeted approaches have been used over the past years, including the anti-VEGF inhibitor (bevacizumab) [4], anti-PD-1 checkpoint inhibitor (pembrolizumab) [5], or an antibody–drug conjugate that targets tissue factors (tisotumab–vedotin (TV-1) [6]). By conducting a comprehensive retrospective analysis of patients’ molecular profiles at a tertiary cancer center, the authors delve into the practical application of precision oncology, revealing both its enormous potential and the significant challenges it faces in real-world settings. The discovery of a significant number of actionable mutations in the patient cohort highlights the promise of tailored treatments, implying a future in which therapy is tailored to the genetic makeup of an individual’s cancer, potentially improving outcomes and reducing side effects. In addition, the study provides a thorough overview of the cases handled by the interdisciplinary Molecular Tumor Board, demonstrating the complex decision-making processes involved and the factors that can cause deviations from recommended precision oncology pathways. Furthermore, this study sheds light on the broader implications of the integration of data science into oncology. It demonstrates how genomic data can guide treatment decisions, pointing to a future in which cancer care is highly personalized. However, it also reflects the systemic and infrastructural changes required to make this future a reality, such as improving the accessibility of targeted treatments and ensuring that healthcare professionals are prepared to interpret and act on complex genomic data.

Furthermore, the study by Liu et al. on hepatocellular carcinoma (HCC) is an excellent example of how data science can significantly improve our understanding of cancer genetics. Genome-wide association studies (GWAS) are utilized to explore genetic associations in various diseases, including HCC. Many studies have identified genetic factors that are associated with the development of this disease and numerous single-nucleotide variations (SNVs) in different genomic regions [7,8]. The authors mapped the previously unknown genetic landscape of HCC using such a method, and they identified thirteen novel genetic loci that may be important in the risk for and development of hepatocellular carcinoma. This endeavor not only broadens the scope of our genetic knowledge about HCC, but also emphasizes the importance of employing large-scale data analysis in modern oncology research. The use of polygenic risk score (PRS) models enhances the significance of this study’s findings, providing a more nuanced approach to understanding individual susceptibility to HCC. Liu et al. demonstrate the universality of their findings by drawing on data from a wide range of populations, including invaluable insights from the UK Biobank and Japanese databases. This methodological inclusivity ensures that the implications of this research are not constrained by geographical or ethnic boundaries, paving the way for the development of universally applicable diagnostic and preventive measures.

In addition, the investigation by Ioannou et al. into kidney cancer through a systems biology approach represents a significant step forward in the understanding and treatment of this complex disease. By meticulously analyzing co-deregulated genes across various studies taken from the Gene Expression Omnibus (GEO) using a new multivariate method called CharacteristicDirection (CD) [9], the authors not only shed light on the molecular intricacies of kidney cancer, but also opened the door to innovative therapeutic strategies. The identification and annotation of co-deregulated genes across different kidney cancer subtypes reveals the diverse molecular mechanisms at play. Through the use of advanced computational tools, the authors dissect the complex networks formed by these genes and their upstream regulators, laying the genetic underpinnings of kidney cancer bare in unprecedented detail. Moreover, by leveraging databases such as CMap, DrugMatrix, and LINCS L1000, the researchers highlight several existing drugs that could potentially reverse the expression of the identified co-deregulated genes, thus offering new hope for kidney cancer treatment. This aspect of the research underscores the practical implications of these findings, demonstrating how computational biology can bridge the gap between gene expression studies and real-world therapeutic applications.

While the expression of androgen receptor (AR) is more prevalent in breast cancer than estrogen receptor (ER) activity [10,11], the detailed molecular role of AR in breast cancer remains unresolved. It has previously been shown that AR supports estradiol-mediated ER activity in ER-positive/AR-positive breast cancer [12]. AR inhibition can be synergized with tamoxifen to reduce the proliferation of ER-positive breast cancer [13]. The study by Long et al. delves into the nuanced interplay between the expression of AR and ER in breast cancer, challenging long-standing classifications and opening up new avenues for the development of patient-specific treatment paradigms. This research illuminates the prognostic value of AR expression across different breast cancer subtypes, revealing that AR positivity could herald a distinct clinical course and response to therapy, irrespective of ER status. By dissecting the role of AR in both ER-positive and ER-negative breast cancers, Long and colleagues not only contribute to a more refined understanding of breast cancer biology, but also underscore the potential for using AR as a biomarker to guide treatment decisions. This study is significant because it has the potential to change the clinical management of breast cancer. Traditionally, breast cancer treatment strategies were heavily reliant on the presence or absence of ER, PR, and HER2. Long et al.’s findings suggest that incorporating the AR status into breast cancer subtyping could improve prognosis and treatment customization. This approach could be especially beneficial for patients with ER-negative breast cancer, where treatment options are limited and prognoses are frequently poor. By identifying a subset of ER-negative breast cancers with positive levels of AR expression, the study points to the possibility of using AR as a therapeutic target, potentially expanding the arsenal of treatments available for this challenging group.

An important issue that was not addressed until recently is the analysis of genes and signaling pathways that are co-deregulated across the molecularly heterogeneous subtypes of lung cancer [14,15]. The analysis of co-deregulated signaling pathways across these subtypes could be extremely useful in their treatment. In their meticulous investigation into lung cancer, Chatziantoniou and Zaravinos dissect the complex molecular landscape of lung cancer through the prism of co-deregulated genes and their intricate networks. By leveraging a robust methodology that combines the analysis of gene expression data with the exploration of drug databases, this study casts new light on the genetic intricacies driving lung cancer progression and responses to therapy. This exploration into the co-deregulated genes not only deepens our understanding of lung cancer’s molecular underpinnings, but also opens up new opportunities for the development of personalized treatment avenues. The significance of this work lies in its potential to revolutionize lung cancer treatment by moving beyond the traditional one-size-fits-all approach. By identifying specific gene networks that are altered across different subtypes of lung cancer, the study underscores the heterogeneity of this disease, advancing a tailored approach to treatment that considers the unique genetic makeup of each tumor. This level of granularity in understanding the molecular drivers of lung cancer could lead to more effective and less toxic therapeutic options, enhancing patient outcomes. Furthermore, the study’s emphasis on drug repurposing based on the identified co-deregulated genes suggests a cost-effective and time-efficient approach to discovering new treatment options.

Diverse research fields are focusing on heterogeneous gene regulatory networks in order to better understand disease mechanisms. Several computational methods have been developed for their estimation. Furthermore, the effectiveness of these gene networks has been validated in a variety of studies, including drug combination identification and cancer prediction projects [16,17]. Nevertheless, most of these studies have used averaged gene networks across cell lines, leading to the inability to identify specific gene regulatory networks. The comprehensive review by Heewon Park is a nice example of the transformative potential of computational biology in terms of investigating gene regulatory networks that underpin the epithelial–mesenchymal transition (EMT) in cancer. EMT plays a crucial role in cancer progression, metastasis, and resistance to therapy, representing a pivotal area of research for developing targeted cancer treatments. Park’s review meticulously explores the nuances of EMT status across different cell lines, shedding light on the molecular dialogues that dictate this complex biological process. By emphasizing the significance of computational strategies, this study delineates a roadmap for leveraging data science to decode the multifaceted nature of cancer progression, thereby advancing the frontier of precision medicine. This review also offers insights into the various methodologies and algorithms that can be employed to estimate cell-line-specific gene regulatory networks. This exploration is crucial for understanding how different EMT statuses can influence cancer behavior, including tumorigenesis, invasion, and metastasis. Park underscores the importance of this understanding for identifying potential therapeutic targets that could inhibit or reverse the EMT process, thus impeding cancer progression. Moreover, the review introduces an explainable artificial intelligence (AI) approach for interpreting the vast quantity of data generated by studying multiple EMT-status-specific gene networks. This aspect of the work is particularly noteworthy as it addresses a significant challenge in computational biology: the interpretation of complex, high-dimensional data in a manner that is both comprehensive and accessible to researchers and clinicians. By employing explainable AI, Park’s review illuminates a path toward not only understanding but also effectively communicating the insights gleaned from computational analyses, thereby enhancing their applicability in clinical settings.

Another comprehensive review by Alizadeh et al. serves as a pivotal exploration into the field of gastroenterology research, spotlighting the revolutionary role of big data and multi-omics approaches in unveiling the multifaceted nature of gastrointestinal diseases. The study shows how the combined use of genomic, transcriptomic, proteomic, and metabolomic data can provide a holistic understanding of the underlying mechanisms that drive various gastrointestinal conditions, including cancer. By advocating for the integration of these multi-omics approaches, this review not only emphasizes the complexity of biological and disease processes, but also showcases the immense potential of data science to transform our approaches to research, diagnosis, and treatment. Interestingly, the significance of this review extends beyond gastroenterology, benefiting the larger scientific community interested in using big data to address complex health issues. The study meticulously outlines the advantages of a holistic omics approach, such as the ability to discover new biomarkers for disease, identify therapeutic targets, and comprehend the molecular basis of disease progression and responses to treatment. This comprehensive perspective is critical for advancing personalized medicine, which allows treatments to be tailored to each individual patient based on a thorough understanding of their unique biological makeup. Furthermore, the review emphasizes the challenges and opportunities that come with integrating big data into healthcare research. The authors discuss potential pitfalls in study design and execution, providing valuable insights into how to navigate the complexities of big data analysis while avoiding common mistakes. This guidance is critical for ensuring that multi-omics research delivers tangible benefits for patient care.

Finally, I would like to thank the authors, reviewers, and Editorial Team for their invaluable contributions to this Special Issue. Taken together, these studies demonstrate data science’s incredible potential to transform cancer genomics and precision medicine. They not only provide invaluable insights into the molecular underpinnings of various cancers, but also lay the groundwork for the next generation of personalized cancer therapies. As we approach a new era in oncology, the contributions in this Special Issue serve as both a testament to our current accomplishments and a roadmap for the future of cancer research and treatment.

## References

[1] Berger M.F., Mardis E.R. (2018). The Emerging Clinical Relevance of Genomics in Cancer Medicine. Nat. Rev. Clin. Oncol..

[2] Mendelsohn J. (2013). Personalizing Oncology: Perspectives and Prospects. J. Clin. Oncol..

[3] Ginsburg G.S., Phillips K.A. (2018). Precision Medicine: From Science To Value. Health Aff. Proj. Hope.

[4] Tewari K.S., Sill M.W., Long H.J., Penson R.T., Huang H., Ramondetta L.M., Landrum L.M., Oaknin A., Reid T.J., Leitao M.M. (2014). Improved Survival with Bevacizumab in Advanced Cervical Cancer. N. Engl. J. Med..

[5] Colombo N., Dubot C., Lorusso D., Caceres M.V., Hasegawa K., Shapira-Frommer R., Tewari K.S., Salman P., Hoyos Usta E., Yañez E. (2021). Pembrolizumab for Persistent, Recurrent, or Metastatic Cervical Cancer. N. Engl. J. Med..

[6] Coleman R.L., Lorusso D., Gennigens C., González-Martín A., Randall L., Cibula D., Lund B., Woelber L., Pignata S., Forget F. (2021). Efficacy and Safety of Tisotumab Vedotin in Previously Treated Recurrent or Metastatic Cervical Cancer (innovaTV 204/GOG-3023/ENGOT-Cx6): A Multicentre, Open-Label, Single-Arm, Phase 2 Study. Lancet Oncol..

[7] Li Y., Zhai Y., Song Q., Zhang H., Cao P., Ping J., Liu X., Guo B., Liu G., Song J. (2018). Genome-Wide Association Study Identifies a New Locus at 7q21.13 Associated with Hepatitis B Virus–Related Hepatocellular Carcinoma. Clin. Cancer Res..

[8] Li S., Qian J., Yang Y., Zhao W., Dai J., Bei J.-X., Foo J.N., McLaren P.J., Li Z., Yang J. (2012). GWAS Identifies Novel Susceptibility Loci on 6p21.32 and 21q21.3 for Hepatocellular Carcinoma in Chronic Hepatitis B Virus Carriers. PLoS Genet..

[9] Clark N.R., Hu K.S., Feldmann A.S., Kou Y., Chen E.Y., Duan Q., Ma’ayan A. (2014). The Characteristic Direction: A Geometrical Approach to Identify Differentially Expressed Genes. BMC Bioinform..

[10] Kensler K.H., Regan M.M., Heng Y.J., Baker G.M., Pyle M.E., Schnitt S.J., Hazra A., Kammler R., Thürlimann B., Colleoni M. (2019). Prognostic and Predictive Value of Androgen Receptor Expression in Postmenopausal Women with Estrogen Receptor-Positive Breast Cancer: Results from the Breast International Group Trial 1–98. Breast Cancer Res..

[11] Hickey T.E., Robinson J.L.L., Carroll J.S., Tilley W.D. (2012). Minireview: The Androgen Receptor in Breast Tissues: Growth Inhibitor, Tumor Suppressor, Oncogene?. Mol. Endocrinol..

[12] D’Amato N.C., Gordon M.A., Babbs B., Spoelstra N.S., Carson Butterfield K.T., Torkko K.C., Phan V.T., Barton V.N., Rogers T.J., Sartorius C.A. (2016). Cooperative Dynamics of AR and ER Activity in Breast Cancer. Mol. Cancer Res..

[13] Okano M., Oshi M., Butash A.L., Asaoka M., Katsuta E., Peng X., Qi Q., Yan L., Takabe K. (2019). Estrogen Receptor Positive Breast Cancer with High Expression of Androgen Receptor Has Less Cytolytic Activity and Worse Response to Neoadjuvant Chemotherapy but Better Survival. Int. J. Mol. Sci..

[14] Wen S., Peng W., Chen Y., Du X., Xia J., Shen B., Zhou G. (2022). Four Differentially Expressed Genes Can Predict Prognosis and Microenvironment Immune Infiltration in Lung Cancer: A Study Based on Data from the GEO. BMC Cancer.

[15] Gao M., Kong W., Huang Z., Xie Z. (2020). Identification of Key Genes Related to Lung Squamous Cell Carcinoma Using Bioinformatics Analysis. Int. J. Mol. Sci..

[16] Daoud M., Mayo M. (2019). A Survey of Neural Network-Based Cancer Prediction Models from Microarray Data. Artif. Intell. Med..

[17] Cheng F., Kovács I.A., Barabási A.-L. (2019). Network-Based Prediction of Drug Combinations. Nat. Commun..

